# The GluA1 AMPAR subunit is necessary for hedonic responding but not hedonic value in female mice

**DOI:** 10.1016/j.physbeh.2020.113206

**Published:** 2021-01-01

**Authors:** Jasmin A. Strickland, Joseph M. Austen, Rolf Sprengel, David J. Sanderson

**Affiliations:** Department of Psychology, Durham University, South Road, Durham DH1 3LE, United States

**Keywords:** Anhedonia, Palatability, Reinforcement, Learning, Consumption, Mice

## Abstract

The GluA1 subunit of the AMPA receptor has been implicated in anhedonia. Mice that lack GluA1 (*Gria1* knockout mice) show reduced lick cluster size, a measure of palatability in feeding behaviour. This deficit may reflect a role for GluA1 in encoding the hedonic value of palatable substances or instead a role for GluA1 in the behavioural expression of hedonic value. We tested the role of GluA1 in hedonic value by assessing sensitivity to changes in the rewarding property of sucrose as a consequence of negative/positive contrast effects in female mice. During training, on half of the days consumption of a flavour (CS+) mixed with 4% sucrose was preceded by consumption of 1% sucrose (positive contrast). On the other half of days consumption of a different flavour (CS–) mixed with 4% sucrose was preceded by consumption of 16% sucrose (negative contrast). In the test session both wild-type, controls and *Gria1* knockout mice consumed more of the CS+ flavour than the CS– flavour. While *Gria1* knockout mice showed reduced lick cluster sizes, both genotypes made larger lick clusters for the CS+ flavour than the CS– flavour suggesting that the CS+ was more palatable than the CS–. A follow up experiment in normal mice demonstrated that the negative contrast procedure resulted in a conditioned reduction of palatability of the CS– in comparison to an associatively neutral, novel flavour. The results failed to demonstrate a role for GluA1 in hedonic value suggesting that, instead, GluA1 is necessary for hedonic responding.

## Highlights

• GluA1 containing AMPA receptors are necessary for palatability responses to sucrose.
• Mice lacking GluA1 showed were sensitive to changes in the hedonic value of sucrose.
• This suggests that GluA1 is necessary for hedonic responding but not hedonic value.

## 1 Introduction

In line with the glutamatergic hypothesis of schizophrenia [1], *Gria1*, the gene that encodes for the GluA1 subunit of the AMPA receptor for glutamate, has genome wide association to schizophrenia [2, 3]. This supports previous post-mortem work that found a reduction in hippocampal GluA1 mRNA[4, 5], and GluA1 [6]and AMPA binding sites [7]in schizophrenia patients. Genetically modified mice that lack *Gria1* provide a means of assessing the role of GluA1 in behaviour relevant to the symptoms of schizophrenia.

GluA1 deletion in mice has been found to mimic some of the negative symptoms of schizophrenia such as anhedonia [8, 9]. In order to test the role of *Gria1* in anhedonia, Austen, Sprengel, and Sanderson [10] examined the effect of GluA1 deletion on consumption of sucrose. *Gria1* knockout (*Gria1*^−/−^) mice showed normal levels of consumption of sucrose when tested over an hour period and showed normal flavour conditioning in which mice consumed more of a flavour previously paired with a high concentration of sucrose compared to a flavour paired with a low concentration. In contrast, lick cluster size, a measure of palatability, was reduced in *Gria1*^−/−^ mice.

Lick cluster size is the number of licks made in quick succession before a pause in licking. Typically, a run of licks made with less than 0.5 s between each lick is considered to reflect a lick cluster [11]. In normal rodents the number of licks in a cluster increases as function of sucrose concentration [11], [12], [13]. This is in contrast to measures of consumption that show an inverted U-shaped function, initially increasing with sucrose concentration, but peaking at intermediate concentrations and progressively reducing with further increases in concentration [12, 14]. The dissociation between lick cluster size and consumption suggests that lick cluster size provides a measure of palatability independent of other effects on consumption. Furthermore, manipulations that affect lick cluster size also typically affect orofacial responses consistent with lick cluster size being a measure of hedonic responding (see [15] for a review).

While reduced lick cluster size in *Gria1*^−/−^ mice suggests that GluA1-containing AMPA receptors play a role in hedonic responding (i.e., the behavioural response to a palatable substance, e.g., lick cluster size), the cause of the impairment is not clear. It is possible that GluA1 is necessary for sensitivity to the hedonic value (i.e., how pleasurable a stimulus is) of sucrose. Consequently, GluA1 deletion may result in a reduction of the palatability of sucrose (i.e., sucrose tastes less sweet than it would otherwise do). Alternatively, GluA1 may be necessary for the behavioural expression of palatability. Therefore, GluA1 deletion may impair lick cluster size (the hedonic response to sucrose) but not necessarily how sweet sucrose tastes (the hedonic value of sucrose).

The results of our previous flavour conditioning experiments provide equivocal evidence for the role of GluA1 in hedonic value. Austen, et al. [10] found that GluA1 deletion spared flavour conditioning that depends on the rewarding effect of sucrose. During the training phase of the experiment, mice consumed a flavour (CS+) mixed with 32% sucrose on half of the training days and a different flavour (CS–) mixed with 4% sucrose on the other half of training days. In the test session, mice were allowed to consume both flavours mixed with 4% sucrose. *Gria1*^−/−^ and wild-type, control mice consumed more of the CS+ flavour than the CS– flavour. While this result suggests that GluA1 is not necessary for learning an association between a flavour and the rewarding properties of sucrose, it is not clear to what extent learning reflects an association between the flavour and the hedonic properties of sucrose or its nutritional properties. Certainly, flavour preference learning can be achieved with intragastric infusions of sucrose [16], suggesting that experience of the palatability of sucrose is not necessary for flavour conditioning with sucrose as the reinforcer.

A potential way to disentangle the role of GluA1 in hedonic value and hedonic responding is to manipulate the palatability of sucrose in a learning procedure that relies on the rewarding properties of sucrose. Dwyer, Figueroa, Gasalla, and Lopez [17] demonstrated that recent experience of sucrose affects the palatability of sucrose such that it can influence the ability of sucrose to support flavour conditioning. Rats drank two flavours that each functioned as a conditioned stimulus (CS). Each flavour was mixed with 8% sucrose (unconditioned stimulus, US) and the flavours were consumed on separate days. During training consumption of one flavour (CS+) was always preceded by consumption of 2% sucrose. Consumption of the other flavour (CS–) was always preceded by 32% sucrose. In the test session, rats were allowed to drink both flavours mixed with 8% sucrose. Even though both flavours had been paired with 8% sucrose during training, rats drank more of the CS+ flavour than the CS– flavour. Furthermore, lick cluster sizes were greater for the CS+ than the CS– flavour. This suggests that during the training phase the level of sucrose paired with the CS+ flavour was perceived as more palatable than that paired with the CS– flavour. Dwyer, et al. [17] interpreted this effect in terms of negative and positive contrast. During training the 8% sucrose solution paired with the CS– was experienced in the context of prior consumption of the more palatable 32% sucrose solution (negative contrast), whereas the 8% sucrose solution paired with the CS+ was experienced in the context of the less palatable 2% sucrose solution (positive contrast). These contrast effects may reflect habituation to sucrose that results in sucrose being relatively more (in the positive contrast condition) or less (in the negative contrast condition) effective as a reinforcer in flavour conditioning.

In order to test whether GluA1 is necessary for hedonic value, in Experiment 1, we tested *Gria1*^−/−^ female mice on a procedure similar to that used by Dwyer, et al. [17]. Impaired sensitivity to hedonic value should result in *Gria1*^−/−^ mice showing similar levels of consumption of the CS+ and CS– flavours. This result would indicate that the reduced lick cluster size in *Gria1*^−/−^ mice [10] is a consequence of reduced hedonic value rather than simply impaired behavioural expression of palatability. If instead it was found that *Gria1*^−/−^ mice showed greater consumption of the CS+ flavour than the CS– flavour in a similar manner to wild-type control mice then this would suggest that hedonic value is preserved in *Gria1*^−/−^ mice and the reduced lick cluster size reflects impaired behavioural expression of palatability.

An additional experiment was performed to rule out a potential confound in Experiment 1. As a consequence of the sucrose adaptation procedure in Experiment 1 we found that mice consumed less of the CS– flavour than CS+ flavour during training. In order to rule out the possibility that differences in the extent of consumption during training affected test performance, an experiment in normal female mice was conducted in which consumption of the CS– flavour at test was compared with consumption of a novel flavour. If performance at test is simply a consequence of the extent of consumption during training then mice should consume more of the CS– than novel flavour. In contrast, greater consumption of the novel flavour compared to the CS– flavour would suggest that the prior experience of the CS– during training led to a reduction in palatability of the CS– flavour.

## 2 Methods

### 2.1 Subjects

Experiment 1 used *Gria1*^−/−^ and wild-type, littermate control female mice (*N* = 13 per genotype), bred and housed in the life sciences support unit at Durham University. Mice were experimentally naïve at the start of testing. Mice were housed in groups of 1–5 in a temperature-controlled housing room with a 12hr light dark cycle (8am-8pm). They were approximately 17–38 weeks old at the start of testing (age range for wild-type: 17–38 weeks; age range for *Gria1*^−/−^ mice: 17–34 weeks). They were maintained at 85% of their free-feeding weights (19.7 g – 28.0 g) with ad libitum access to water in their home cages. Experiment 2 used 16 experimentally naïve female C57BL6J mice purchased from Charles River, UK. Mice were housed in cages of four mice. They were approximately 10 weeks old at the starting of training. Their free-feeding weight was 16.7–20.5 g. All other details were the same as for Experiment 1.

### 2.2 Apparatus

Eight identical operant chambers (interior dimensions: 21.6 × 17.8 × 12.7 cm; ENV-307 W, Med Associates, Inc., Fairfax, VT, USA), enclosed in sound-attenuating cubicles (ENV-022 V, Med Associates) were used. The chambers were controlled by Med-PC IV software (Med Associates). The side walls were made from aluminium and the front and back walls and the ceiling were made from clear Perspex. The chamber floors each comprised a grid of 24 stainless steel rods (0.32 cm diameter), spaced 0.79 cm apart and running perpendicular to the front of the chamber (ENV-307W-GFW, Med Associates). A fan (ENV-025F, Med Associates) was located within each of the cubicles and was turned on during sessions. Retractable sippers (ENV-352AW, Med Associates) and a small hole in one wall of each chamber allowed sipper tubes to be extended into, and retracted from, the chambers. Contact lickometer controllers (ENV-250, Med Associates) allowed contacts between the mice and the sipper tubes to be recorded at a resolution of 0.01 s. Sucrose solutions were made w/v with commercially available sucrose in distilled water. Cherry and grape Kool-Aid, 0.05%wt/vol, (Kraft Foods., Rye Brook, NY) were used as the CS+ and CS– flavours.

### 2.3 Procedure

#### 2.3.1 Experiment 1

In each daily session mice had two 10-minute periods of access to the sipper tube separated by an interval of approximately 10 min in which mice were returned to the home cage. On half of the sessions, mice had 10 min of access to 1% sucrose (with no flavour) and then 10 min of access to the CS+ flavour mixed with 4% sucrose. On the other half of sessions, mice had 10 min of access to 16% sucrose (with no flavour) followed by 10 min of access to the CS–flavour mixed with 4% sucrose. Mice received eight days of training, four with each flavour. Flavours were presented in a double alternating order (i.e., XYYX) across days. On the ninth day mice received a test session. Similar to training sessions, mice received two 10-min periods of access to the sipper tube. The CS– and CS+ flavours mixed with 4% sucrose were presented, one in each 10-minute period of access. The allocation of flavours to conditions (CS+ or CS–), the order of flavours and conditions during training and within the test session was counterbalanced within genotype and sex as far as possible given the number of mice within each subgroup.

### 2.3.2 Experiment 2

The training phase of Experiment 2 was similar to that for Experiment 1, but the CS+ training days were omitted. Therefore, mice received only four days of training in which they consumed 16% sucrose followed by the CS– flavour mixed with 4% sucrose. One day after the last training day mice received a test session in which they were allowed to consumed the CS– flavour and a novel flavour both mixed with 4% sucrose. These flavours were administered in a similar manner as the test day in Experiment 1. The allocation of flavours (cherry and grape) across conditions (CS–, novel) was counterbalanced across mice as was the order of the conditions (CS–, novel) and flavours (grape, cherry) within the test session.

### 2.4 Data analysis

In each session the total number of licks, the mean lick cluster size, and the amount consumed were recorded. Amount consumed was measured by weighing the sipper tube before and after each period of access. Lick clusters were defined as two or more licks made with less than 0.5 s between the end of one lick and the start of the next. Once the inter-lick interval was 0.5 s or longer, the lick cluster was deemed to have ended. The mean lick cluster size was calculated by dividing the total number of licks that were made within clusters of licks by the number of completed lick clusters. If a mouse was midway through a lick cluster when the tube was retracted at the end of the period of access to the sipper tube the licks were added to the total number of licks made within lick clusters, but no completed cluster was counted. Therefore, this method potentially over estimates lick cluster size to a small degree.

### 2.5 Statistical analysis

For the training phase of Experiment 1, separate analyses were conducted for the first period of access (1% and 16% sucrose) and the second period of access (CS+ and CS–). Analyses used ANOVA with genotype, session, and solution (i.e., 1% vs. 16% sucrose or CS+ vs. CS–) as factors. The test data were analysed using ANOVA with genotype and CS (CS+ and CS–) as factors. For Experiment 2 one-way ANOVAs were conducted separately for consumption of 16% sucrose and the CS– flavour during training. The test phase data were analysed using paired-sample t-tests. When sphericity of within-subjects variables with more than two levels could not be assumed, a Greenhouse-Geisser correction was applied to produce more conservative p-values. The non-corrected degrees of freedom are reported. For t-values we report Cohen's d for the effect size with 95% confidence intervals. For F-values we report η_p_^2^ for the effect size with 90% confidence intervals. 90% confidence intervals that exclude zero indicate that the F-value is significant at *p*< .05 [18].

## 3 Results

### 3.1 Experiment 1

#### 3.1.1 Training

##### 3.1.1.1 Consumption

**Fig. 1:**
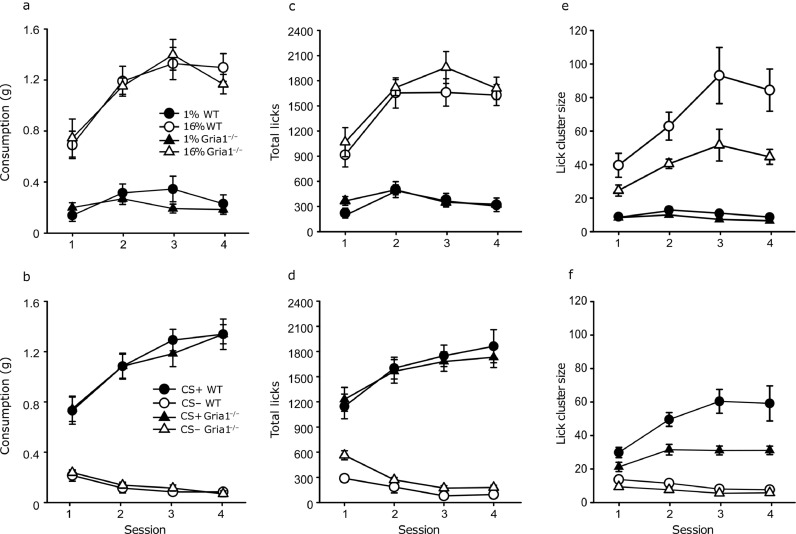
Mean consumption (g), total numbers of licks and lick cluster sizes during the first periods of access to the 1% or 16% sucrose solutions (panels a, c and e respectively) and in the second periods of access to the CS+ or CS- flavoured 4% sucrose solutions (panels b, d and f respectively) for control wild-type (WT) and *Gria1*^–/–^ mice in Experiment 1. Error bars show ±SEM.

In the first period of access consumption of 16% sucrose was greater than 1% sucrose (Fig. 1a, F(1,24) = 406.23, *p*< .001, η_p_^2^ = 0.944, 90% CI[.89,.96]). There was no significant difference in the volumes consumed by wild-type and *Gria1*^–/–^ mice (*F*< 1, *p* = .63). There was a significant effect of session, F(3,72) = 15.95, *p*< .001, η_p_^2^ = 0.39, 90% CI[.23,.49] and a significant interaction between sucrose concentration and session, F(3,72) = 8.11, *p*< .001, η_p_^2^ = 0.25, 90% CI[.09,.35]. All other interactions were not significant (F-values < 1, p-values >0.61). In the second period of access (Fig. 1b) consumption of the CS+ was greater than the CS–, F(1,24) = 380.65, *p*<0.001, η_p_^2^ = 0.94, 90% CI[.89,.95]. The amount consumed did not significantly differ between the wild-type and *Gria1*^–/–^ mice, *F*< 1, *p* = .94. There was a significant effect of session, F(3,72) = 11.45, *p*< .001, η_p_^2^ = 0.32, 90% CI[.15,.42] and a significant interaction between flavour and session, F(3,72) = 34.30, *p*< .001, η_p_^2^ = 0.58, 90% CI[.44,.66]. All other interactions were not significant, F-values < 1, p-values >0.77.

##### 3.1.1.2 Total licks

In the first period of access both wild-type and *Gria1*^–/–^ mice made more licks for 16% sucrose than 1% sucrose (see Fig. 1c). There was a significant effect of sucrose concentration, F(1,24) = 487.51, *p*< .001, η_p_^2^ = 0.95, 90% CI[.90,.96] no significant effect of genotype, F(1,24) = 1.03, *p* = .31, a significant effect of session, F(3,72) = 15.93, *p*< .001, η_p_^2^ = 0.39, 90% CI[.23,.49] and a significant interaction between session and sucrose concentration F(3,72) = 9.08, *p*< .001, η_p_^2^ = 0.27, 90% CI[.11,.38]. All other interactions were not significant, F-values < 1.2, p-values >0.27. In the second period of access (Fig. 1d) both groups made a greater number of licks during consumption of the CS+ than the CS–, F(1,24) = 348.13, *p*< .001, η_p_^2^ = 0.93, 90% CI[.88,.95]. There was no significant effect of genotype, *F*< 1, *p* = .58. There was a significant effect of session F(3,72) = 2.83, *p* = .44, η_p_^2^ = 0.10, 90% CI[.001,.19], and significant interaction between flavour and session, F(3,72) = 29.68, *p*< .001, η_p_^2^ = 0.55, 90% CI[.40,.63]. All other interactions were not significant, F-values < 1.4, p-values >0.29.

##### 3.1.1.3 Lick cluster size

In the first period of access mean lick cluster sizes were significantly greater during consumption of the 16% than the 1% (Fig. 1e, F(1,24) = 108.28, *p*< .001, η_p_^2^ = 0.81, 90% CI[.67,.87]) and were significantly reduced in the *Gria1*^–/–^ mice, F(1,24) = 12.67, *p* = .002, η_p_^2^ = 0.34, 90% CI[.09,.52]. The effect of sucrose concentration was greater in wild-type mice than *Gria1*^–/–^ mice (genotype by sucrose concentration interaction, F(1,24) = 9.63, *p* = .005, η_p_^2^ = 0.28, 90% CI[.059,.47]). There was a significant effect of session, F(3,72) = 9.63, *p*< .001, η_p_^2^ = 0.28, 90% CI[.12,.39] and a significant sucrose concentration by session interaction, F(3,72) = 10.24, *p*< .001, η_p_^2^ = 0.29, 90% CI[.13,.40]. All other interactions were not significant, F-values < 1.5, p-values >0.21. In the second period of access (Fig. 1f) mean lick cluster sizes were greater during consumption of the CS+ than the CS–, F(1,24) = 190.76, *p*< .001, η_p_^2^ = 0.88, 90% CI[.79,.91]. Mean lick cluster sizes were significantly reduced in the *Gria1*^–/–^ mice compared to wild-type mice, F(1,24) = 28.32, *p*< .001, η_p_^2^ = 0.54, 90% CI[.28,.67]. The effect of CS on lick cluster size was greater in wild-type mice than *Gria1*^–/–^ mice (CS flavour by genotype interaction, F(1,24) = 16.54, *p*<0.001, η_p_^2^ = 0.408, 90% CI[.14,.57]). There was a significant effect of session, F(3,72) = 4.68, *p* = .005, η_p_^2^ = 0.16, 90% CI[.03,.26] and a significant interaction between session and flavour, F(3,72) = 10.76, *p*< .001, η_p_^2^ = 0.31, 90% CI[.14,.41]. All other interactions were not significant (F-values < 2.2, p-values >0.094).

#### 3.1.2 Test

##### 3.1.2.1 Consumption

**Fig. 2:**
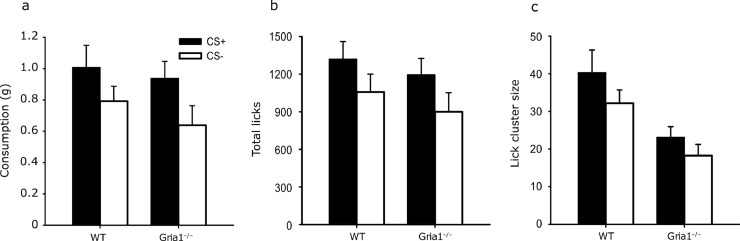
Mean consumption (panel a), total numbers of licks (panel b) and lick cluster size (panel c), for the CS+ and CS– during the test session for wild-type (WT) and *Gria1*^–/–^ mice in Experiment 1. Error bars show SEM.

Both wild-type and *Gria1*^‒/–^ mice consumed more of the CS+ than the CS– (Fig. 2a, F(1,24) = 7.76, *p* = .01, η_p_^2^ = 0.24, 90% CI[.036,.44]). There was no significant effect of genotype, *F*< 1, *p* = .43, or interaction between cue and genotype, F(1,24) = 2.09, *p* = .16.

##### 3.1.2.2 Total licks

Both wild-type and *Gria1*^–/–^ mice made more licks during consumption of the CS+ than the CS– (Fig. 2b, F(1,24) = 5.24, *p* = .031, η_p_^2^ = 0.17, 90% CI[.009,.38]). There was no significant effect of genotype, *F*< 1, *p* = .46, or significant interaction between flavour and genotype (*F*< 1, *p* = .89).

##### 3.1.2.3 Lick cluster size

Although both wild-type and *Gria1*^‒/‒^ mice made numerically larger lick cluster sizes during consumption of the CS+ than the CS–, this failed to reach significance (Fig. 2c, F(1,24) = 3.52, *p* = .073, η_p_^2^ = 0.12, 90% CI[.00,.32]). Mean lick cluster sizes were significantly reduced in the *Gria1*^‒/‒^ mice, F(1,24) = 11.70, *p* = .002, η_p_^2^ = 0.32, 90% CI[.085,.51]. There was no significant interaction between cue and genotype, *F*< 1, *p* = .63.

### 3.2 Experiment 2

#### 3.2.1 Training

One mouse failed to consume the 16% sucrose or the CS– over the four days of training and was, therefore, removed from the data analysis. Due to equipment failure the licking data for one mouse was lost for consumption of 16% sucrose on the first day of training. Therefore, the data for this mouse was removed from the analysis of total licks and lick cluster size for consumption of 16% during training. The consumption data for this mouse was saved.

##### 3.2.1.1 Consumption

**Fig. 3:**
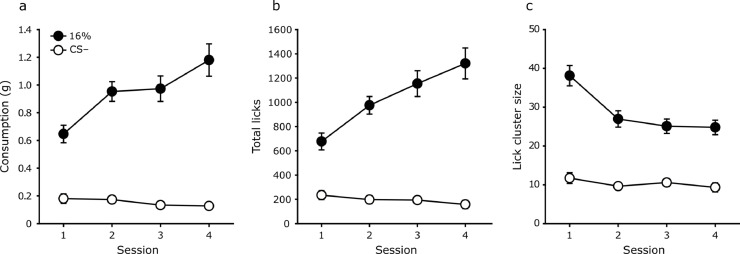
Mean consumption (panel a), total licks (panel b) and lick cluster size (panel c) for 16% sucrose and the CS– over the four sessions of training of Experiment 2. Error bars indicate ±S.E.M.

Consumption for the four days of training is shown in Fig. 3a. Consumption of 16% sucrose increased over days, F(3,42) = 19.46, *p*< .001, η_p_^2^ = 0.58, 90% CI[.38, 0.67]. Consumption of the CS– tended to decrease, but not significantly, F(3,42) = 1.09. *p* = .36.

##### 3.2.1.2 Total licks

The number of licks over the four days of training is shown in Fig. 3b. The pattern for the total licks measure was similar to consumption. Mice increased licking over days for 16% sucrose, F(3,39) = 23.50, *p*< .001, η_p_^2^ = 0.64, 90% CI[.45, 0.72] but not for the CS–, F(3,42) = 1.43, *p* = .25.

##### 3.2.1.3 Lick cluster size

The lick cluster sizes over the four days of training are shown in Fig. 3c. Lick cluster sizes decreased over days for 16% sucrose, F(3,39) = 17.54, *p*< .001, η_p_^2^ = 0.57, 90% CI[.36, 0.66], but were relatively stable for the CS– (F(3,42) = 1.02, *p* = .40.

#### 3.2.2 Test

##### 3.2.2.1 Consumption

**Fig. 4:**
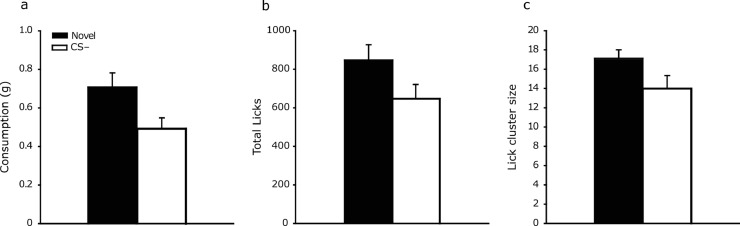
Mean consumption (panel a), total licks (panel b) and lick cluster size (panel c) for the novel and CS– flavours in the test phase of Experiment 2. Error bars indicate S.E.M.

Mice consumed significantly more of the novel flavour than the CS– flavour (see Fig. 4a, t(14) = 2.71, *p* = .017, *d* = 0.70, 95% CI [0.12, 1.26]).

##### 3.2.2.2 Total licks

Mice made a significantly greater number of licks for the novel flavour than CS– flavour (see Fig. 4b, t(14) = 2.48, *p* = .027, *d* = 0.64, 95% CI [0.07, 1.19]).

##### 3.2.2.3 Lick cluster size

Lick cluster sizes were also significantly larger for the novel flavour than CS– flavour (see Fig. 4c, t(14) = 2.40, *p* = .031, *d* = 0.62, 95% CI [0.06, 1.17]).

## 4 General discussion

Previous work found that deletion of GluA1 impairs lick cluster size, a behavioural measure of palatability, suggesting that GluA1 is necessary for hedonic reactions [10]. The purpose of the present study was to test whether the deficit in hedonic responding in female *Gria1*^−/−^mice is due to GluA1 being necessary for representing hedonic value. In the test phase mice consumed more of a flavour paired with 4% sucrose (CS+) that, during the training phase, had been preceded by consumption of 1% sucrose compared to another flavour paired with 4% sucrose (CS−) that had been preceded by 16% sucrose. This suggests that the prior consumption of 1% and 16% sucrose affected the rewarding properties of sucrose such that the CS+ flavour was perceived as being paired with a stronger concentration of sucrose than the CS−. Importantly, *Gria1*^−/−^mice showed greater consumption of the CS+ flavour compared to the CS− flavour to a similar extent as wild-type, control mice. Therefore, this suggests that GluA1 is not necessary for sensitivity to the hedonic value of sucrose that is determined by the extent of recent consumption.

Mice also showed larger lick cluster sizes for the CS+ flavour compared to the CS− flavour. Lick cluster size increases monotonically with sucrose concentration [11], [12], [13]. Therefore, consumption of 16% sucrose before the CS− flavour and 1% sucrose before the CS+ flavour had a conditioned effect on lick cluster size that was similar to manipulating the actual concentration of sucrose. Although GluA1 deletion significantly reduced lick cluster size during the test phase, both *Gria1*^−/−^and wild-type mice showed reduced lick clusters for the CS− flavour compared to the CS+ flavour and there was no significant interaction between factors.

The fact that GluA1 deletion impaired lick cluster size, but did not impair the flavour conditioning that occurred as a consequence of recent sucrose consumption, suggests that GluA1 is necessary for the behavioural expression of palatability, but not learning that depends on the palatability of sucrose. Therefore, GluA1 deletion does not affect the hedonic value of sucrose, but instead affects hedonic responding. *Gria1*^−/−^mice showed reduced lick cluster sizes during the training and test phases of the experiments. Despite this, *Gria1*^−/−^and wild-type mice both showed less consumption of the CS− flavour than the CS+ flavour.

Any conditioned effect of pairing sucrose with a flavour may depend on the palatability of sucrose or its nutritional properties. However, the flavour conditioning effect observed in Experiment 1, which occurred as a consequence of recent sucrose consumption likely depends on the perceived palatability of sucrose rather than any manipulation of its nutritional properties. During training, the CS− was always consumed after recently consuming 16% sucrose, whereas the CS+ was consumed after consuming 1% sucrose. Therefore, the CS− was always experienced in the context of greater energy intake than the CS+. This manipulation also led to differences in satiation and mice consumed less of the CS− than the CS+. As discussed by Dwyer, et al.[17], flavours experienced when sated are subsequently preferred to flavours that have previously been experienced when not sated [19, 20]. Therefore, the fact that mice consumed more of the CS+ than CS− flavour, rather than the opposite pattern, suggests that the conditioned effect is likely to reflect differences in perceived palatability of the US during training, rather than being a consequence of differing levels of satiety.

The conclusions drawn so far are potentially undermined by the fact that consumption of the CS+ was greater than the CS− during training. This was likely due to consumption of 16% sucrose that preceded consumption of the CS− having a greater effect on satiety than consumption of 1% sucrose that preceded the CS+. All other things equal, greater consumption of the CS+ than CS− means that mice had greater experience of the pairing of the CS+ flavour with the US (4% sucrose) compared to the CS−. Therefore, the difference in consumption at test may simply reflect the differences in the extent of the pairing of the CSs with the US. Furthermore, independent of an effect on conditioning, it is also possible that differences in consumption during training resulted in the CS+ being relatively more familiar than the CS− flavour and, therefore, there was greater reduction of neophobia for the CS+ than the CS−.

In order to rule out differences in the amount of consumption of the CS+ and CS− as a cause of the greater consumption of the CS+ during the test phase we conducted Experiment 2. In this experiment, mice received training in which consumption of the CS− was preceded by 16% sucrose. In the test phase, mice were allowed to consume the CS− flavour and a novel flavour. If the extent of exposure to the CS or the CS-US pairing determines levels of consumption at test then mice should consume more of the CS− than the novel flavour. This was found not to be the case and mice showed the opposite pattern, consuming more of the novel flavour than the CS− flavour. Therefore, the extent of experience of the CS-US pairing and the familiarity of the CS is unlikely to account for the results of Experiment 1. The results of Experiment 2 suggest instead that consumption of 16% sucrose prior to consumption of the CS− resulted in a conditioned effect that subsequently led to avoidance of the CS− flavour in comparison to an associatively neutral novel flavour. Furthermore, lick cluster sizes were larger for the novel flavour than the CS– flavour suggesting that the negative contrast procedure resulted in a conditioned reduction in palatability of the CS– flavour.

Avoidance of the CS− flavour may occur because the prior consumption of 16% sucrose led to the 4% sucrose with which the CS− flavour was paired being perceived as weaker than 4%. Subsequently at test, in which both the CS− and novel flavours are mixed with 4% sucrose, the CS− may retrieve a memory of weak sucrose that leads to the CS− being perceived as less sweet than the novel flavour. If this is the case then simply pairing a CS flavour with a low concentration of sucrose during training should, at test, lead to avoidance of that flavour compared to a novel flavour when both flavours are mixed with a higher concentration of sucrose. We have conducted an unpublished test of this account, but have failed to find support for this hypothesis. Mice were allowed to consume a flavour mixed with 1% sucrose during training and then at test the trained flavour and novel flavour were presented mixed with 4% sucrose. Mice showed similar levels of consumption of the trained and novel flavours. Another possibility is that during training 16% sucrose did not just reduce the palatability of the US (4% sucrose), but led to a negative emotional reaction to the 4% sucrose potentially reflecting the frustrative effect of the perceived reduction in the palatability of the US [21]. Which account is more valid remains to be seen, but it is clear that prior consumption of 16% sucrose was sufficient to cause a negative, avoidance and aversion response to the CS− flavour.

The conclusions that can be drawn about the results of Experiment 1 based on the findings of Experiment 2 are potentially limited by the fact that there were differences between the mice used in the two experiments (age, location of breeding etc.). Although we do not know to what extent differences in consumption of the CS+ and CS– during training influenced the test results of Experiment 1, the results of Experiment 2 in which mice consumed more of, and made larger lick clusters for, a novel flavour compared to the trained CS– flavour, at the least show that when the effects of negative contrast and familiarity of flavours are put in competition, the negative contrast effect is greater than any potential familiarity effect.

The results of Experiment 1 demonstrate that *Gria1*^−/−^ mice were sensitive to the effect of recent sucrose exposure on subsequent learning about sucrose. This result is somewhat surprising given that GluA1 deletion has been found to impair short-term habituation to stimuli such that recent experience of a stimulus fails to cause a reduction in subsequent unconditioned responding to that cue [22]. An impairment in short-term habituation in *Gria1*^−/−^ mice has been found with exploration of spatial locations [23, 24] and objects [25], and suppression of food seeking behaviour with visual cues [26]. Although habituation was not measured directly, the results of Experiment 1 suggest that *Gria1*^−/−^ mice did habituate to sucrose as a consequence of consuming 16% sucrose. It is possible that GluA1 deletion impairs the expression of habituation, but not the underlying changes in the perceived intensity/salience of the stimulus. While this is possible this account is at odds with other studies that suggest that GluA1 deletion does affect the perceived salience of stimuli [27, 26]. It is possible that GluA1 is not necessary for habituation to all stimuli.

**Fig. 5:**
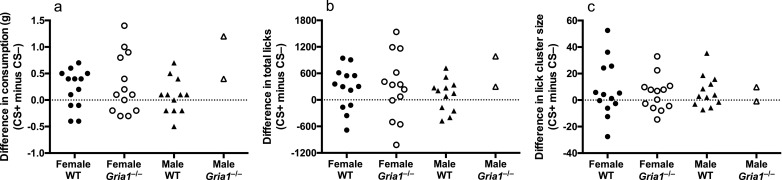
Test phase performance of individual mice separated by sex and genotype for each measure (panel a: consumption (g); panel b: total licks; panel c: lick cluster size). Test performance is shown as the difference between performance for the CS+ and for the CS– (e.g., consumption of the CS+ flavour minus consumption of the CS– flavour). A difference greater than zero indicates that a mouse showed the conditioning effect (i.e., greater consumption and total licks and larger lick cluster size for the CS+ flavour than the CS– flavour).

The effect of GluA1 deletion on hedonic value was tested in female mice. Therefore, we are cautious about drawing conclusions about the role of GluA1 independent of the factor of sex. We did not anticipate an effect of sex, however, because sex did not interact with genotype in our previous analyses of the effect of GluA1 deletion on palatability [10]. The female mice reported in Experiment 1 were actually part of a larger sample run on the procedure in Experiment 1 that included their male siblings (12 wild-type and two *Gria1*^−/−^mice). The data from the male mice were not included in the main analysis presented in the results because of the low numbers of male *Gria1*^−/−^mice (*N* = 2). The test data for all mice (male and female), however, are shown in Fig. 5. Test performance is shown as the difference between performance for the CS+ and for the CS– (e.g., consumption of the CS+ flavour minus consumption of the CS– flavour). A difference greater than zero indicates that a mouse showed the conditioning effect (i.e., greater consumption and total licks and larger lick cluster size for the CS+ flavour than the CS– flavour). While we cannot draw any conclusions about the role of GluA1 in hedonic value in male mice, it was clear that the strength of the flavour conditioning effect was similar between male and female wild-type mice for all measures (consumption, total licks and lick cluster size, see Fig. 5; effect of cue (CS+ versus CS–): F-values < 1, p-values > 0.3).

In conclusion, it is clear that despite an impairment in lick cluster size, *Gria1*^−/−^ mice were sensitive to changes in the hedonic value of sucrose as manipulated by a contrast effect. This suggests that GluA1 is necessary for hedonic responding, but not for hedonic value, potentially limiting the use of *Gria1*^−/−^ mice as a model of the negative symptoms of neuropsychiatric disorders.
